# Metabolomic response of *Zizania latifolia* to low-temperature stress and identification of the bZIP transcription factor family

**DOI:** 10.1080/21645698.2025.2510715

**Published:** 2025-06-16

**Authors:** Yushi Jiang, Xing Chen, Fanxi Wang, Xinze Li, Zhenyuan Qin, Shuang Fan, Ning Yan, Yanning Xie, Rengui Zhao

**Affiliations:** a College of Agronomy, Jilin Agricultural University, Changchun, China; b Key Laboratory of Synthetic Biology of Ministry of Agriculture and Rural Affairs, Tobacco Research Institute, Chinese Academy of Agricultural Sciences, Qingdao, China

**Keywords:** Chinese wild rice (*Zizania latifolia*), low temperature, rice (*oryza sativa*), ZlbZIP transcription factor

## Abstract

Cold stress severely impacts crop production, making it crucial to dissect the metabolic and transcriptional regulatory mechanisms of cold-resistant plants for breeding cold-tolerant varieties. This study systematically explored the response mechanism of *Zizania latifolia* to cold stress by integrating widely targeted metabolomics and genome-wide analysis for the first time. Metabolomics analysis revealed that 690 out of 810 metabolites showed significant differences after cold treatment at 4°C, with significant enrichment of flavonoids, amino acid derivatives, and alkaloids, involving key pathways such as antioxidant defense, osmotic adjustment, and signal transduction. This indicates that *Z. latifolia* copes with cold stress through the coordination of secondary and primary metabolism. A total of 115 bZIP transcription factors (ZlbZIPs) were identified from the *Z. latifolia* genome, with 18 genes located in known cold-resistant quantitative trait locus (QTL) intervals. Four cold-tolerant candidate genes were screened through collinearity analysis with the rice genome. Expression analysis showed that *ZlbZIP005, ZlbZIP075*, and *ZlbZIP084* were significantly upregulated (29.17–4.10 fold) at 24 hours of cold treatment, and their promoter regions with high-density G-box elements implied strong cold response potential. Phylogenetic and evolutionary analyses showed that the bZIP family of *Z. latifolia* is highly homologous to that of rice but exhibits subfamily-specific expansion (such as subfamily Ⅶ) and conserved motif variations related to functional differentiation. This study first elucidated the metabolic reprogramming and bZIP transcription factor regulatory network of *Z. latifolia* under cold stress. The screened key cold-tolerant genes provide important genetic resources for cold-resistant breeding of gramineous crops and lay a foundation for analyzing the molecular mechanism of plant cold resistance and genetic improvement.

## 1. Introduction

Temperature is one of the key environmental factors affecting plant growth and development.^1^ In recent years, the frequency of extreme climate events globally has increased, and low-temperature stress has become one of the major factors limiting agricultural production.^2^ It is estimated that in China alone, low temperatures cause a reduction in grain production of 3 to 5 million tons annually.^3^ With the continuous growth of the global population and the drastic changes in the environment, food security issues have become increasingly prominent. Therefore, the cultivation of high-yielding and cold-resistant crops has become particularly important. In-depth exploration of plant responses to low temperatures, especially the cold tolerance mechanisms of cold-resistant plants, is of significant theoretical and practical value for the development of cold-tolerant crop varieties and the enhancement of crop cold resistance. Low temperature stress severely affects plant growth and survival. When plants are exposed to low temperatures, a series of changes occur in their external morphology and internal physiological and biochemical metabolism.^4,5^

Low-temperature stress can be divided into chilling injury (above 0°C) and freezing injury (below 0°C). Both types of stress adversely affect crop yield and quality because they inhibit various important physiological and biochemical reactions in plants. These changes involve the stability of the cell membrane system, the activity of the antioxidant system, the regulation of fatty acid content, and the accumulation of osmotic regulatory substances.^6^ Additionally, low temperatures can suppress plant respiration and metabolism, reduce root water uptake, and lead to yellowing of leaves and browning of fruits.^7,8^ Low-temperature stress can also lead to the closure of stomata, thereby inhibiting photosynthesis and transpiration activity.^9^ The cell membrane, as the external barrier of plant cells, is highly sensitive to temperature changes. Low temperatures can cause changes in the composition and phase state of membrane lipids, transforming from a flexible liquid crystal state to a harder gel state, thereby reducing membrane fluidity and potentially damaging its function.^10^ This reduction in fluidity affects the exchange of substances and signal transduction between the inside and outside of the cell, thereby disrupting intracellular metabolic processes.^11^ The activity of enzymes on the cell membrane and those associated with the membrane may also be affected by low temperatures, impacting the normal physiological functions of the cell.^12^

In the natural environment, temperature fluctuations are an inevitable challenge during plant growth and development. Plants have evolved various mechanisms over long periods of adversity, enabling them to rapidly and accurately discern various information and respond accordingly, thus adapting well to adverse conditions and ensuring normal plant growth and development.^13^ To adapt to and resist low-temperature stress, plants have evolved complex mechanisms, including the accumulation of certain metabolites with cell protective functions through different means. Especially under cold climatic conditions, plants have evolved a series of complex mechanisms to combat cold. When cold stress occurs, plants need to go through a complex and diverse signal transduction process from sensing low-temperature stimuli to generating responses to cold stress.^14^ In this signaling process, firstly, the low-temperature signal is transmitted between cells, and the formation of the intercellular signal is followed by the conversion of the supramembrane signal. Subsequently, changes in substances on the cell membrane transmit the signal to the active sites of receptor cells. Following intracellular signal transduction, the reversible phosphorylation process of related functional proteins is regulated, allowing the signal to be further conducted within the cell. This induces the production of corresponding regulatory factors that optimize and regulate physiological, biochemical, and molecular functions, thereby enhancing the plant’s resistance to stress.^15,16^ Metabolite accumulation is regulated by the transcriptional reprogramming of a broad spectrum of structural and regulatory genes.^17^ The transcriptional response bridges the perception of upstream low-temperature signals and the expression of downstream specific proteins, constituting one of the main molecular components that coordinate plant cold stress responses and tolerance. Transcription factors play a significant role in the regulation of plant stress responses.^18^

Transcription factors (TFs) are members of a regulatory protein family that, on one hand, bind to cis-acting elements in the promoter regions of different genes, and on the other hand, interact with other TF family members or regulatory proteins to activate or repress the transcription of genes by RNA polymerase, thereby regulating specific physiological and biochemical processes within an organism.^19,20^ When plants are subjected to low-temperature stress, from sensing the low-temperature signal to triggering a series of physiological- and biochemical-level and molecular-level responses, and then generating cold resistance, transcription factors, as an important part of the whole regulatory network, both upwardly undertake the sensing of low-temperature signals and downwardly connect to the expression of cold-resistant specific proteins.^21^ The basic-region leucine zipper (bZIP) transcription factors, which are widely distributed in plants, play crucial roles in plant responses to low-temperature stress and in the regulation of secondary metabolism, including the biosynthesis of terpenoids, alkaloids, and flavonoids.^22^ The basic region, located at the N-terminus of the domain, is not only responsible for binding to cis-acting elements on DNA, determining the specificity of DNA binding, but also contains a nuclear localization signal sequence. This region is more conserved relative to the leucine zipper region.^23^ The leucine zipper motif is characterized by a sequence of seven or nine amino acids that repeat, with leucine typically positioned at the seventh or ninth residue. However, this position can also be occupied by other hydrophobic amino acids, including isoleucine, valine, phenylalanine, or methionine.^24^ The periodic alignment of these hydrophobic amino acids facilitates the formation of protein dimers within this region, which then engage with DNA.^24^ The N-terminus of the bZIP domain, known as the basic region, interacts with the major groove of the DNA helix, while the C-terminus, or leucine zipper region, stabilizes the α-helical structure through dimer formation, a structure referred to as the leucine zipper domain. This domain enables bZIP transcription factors to specifically recognize and bind to particular DNA sequences, thereby regulating the expression of downstream genes.^25,26^

With the increasing attention to the bZIP transcription factor family by researchers, genes encoding for this family have been comprehensively identified and predicted at the whole-genome level in various plants. For instance, 75 genes have been identified in Arabidopsis, 55 in grape, 89 in rice, 131 in soybean, 92 in sorghum, 125 in maize, and 75 in alfalfa.^27^ The induction of genes encoding bZIP proteins by low temperature is widespread in higher plants, and genes capable of encoding bZIP proteins, such as maize mlip15, rice *lip19* and *OsAB15*, and tomato *LebZIP1*, are capable of being expressed by low-temperature control^28–30^; Transformation of Arabidopsis thaliana with soybean *GmbZIP 44, GmbZIP 46, GmbZIP 62*, and *GmbZIP 78* genes has resulted in enhanced salt tolerance and cold resistance in the plants.^31^ Additionally, the expression of 53 *Brassica rapa* bZIP genes undergoes significant changes in response to low-temperature treatment.^32^ At least one-third of the soybean bZIP transcription factors have been demonstrated to participate in cold stress defense. After transferring genes such as *GmbZIP44* into Arabidopsis and inducing their expression under low-temperature conditions, the cold tolerance of the transgenic plants was found to be enhanced compared to the control.^31^ Under cold stress, the rice bZIP transcription factors *OsbZIP73* and *OsbZIP71* form heterodimers, and their co-expression enhances grain filling and seed setting rates. Moreover, the *OsbZIP71/73* heterodimer not only reduces the ABA content in anthers but also increases the transfer of sugars from anthers to pollen, thereby improving seed setting rate, pollen fertility, and rice grain yield.^33^ Furthermore, the early selection of *OsbZIP73* has been shown to enhance the adaptation of japonica rice to cold climates.^34^ Meanwhile, the rice bZIP transcription factor *OsbZIP52* is strongly induced by low temperatures, and its overexpression significantly increases sensitivity to cold stress, suggesting that *OsbZIP52/RISBZ5* acts as a negative regulator in the cold stress response in rice.^35^ Through further research and utilization of these transcription factors, scientists hope to enhance plants’ adaptability to adverse environmental conditions such as low temperatures, thereby improving the sustainability of agricultural production.

Chinese wild rice (*Zizania latifolia*, 2n = 34) is a perennial hydrophyte belonging to the Oryzeae Dum. tribe, Oryzoideae Care subfamily, and Gramineae family.^36^ Its caryopsis is known as Chinese wild rice and has been used as a food grain for more than 3000 years in China.^37^ Not only does Chinese wild rice have high cultivation benefits and significant economic value, but it also primarily grows in lakes and wetlands, possessing an innate advantage of not competing with grain for arable land.^36^ Researchers have employed third-generation sequencing technology to sequence and construct the genome of *Zizania latifolia* (*Z. latifolia*) at the chromosomal level. A total of 545.36 Mb of genomic sequence, accounting for 99.63%, has been anchored to 17 chromosomes, with the corresponding number of sequences being 300. The longest sequence spans 49.61 Mb, while the shortest is 17.01 Mb.^38^ The genomes of *Z. latifolia* and rice exhibit a high degree of collinearity, and phylogenetic analysis indicates that *Z. latifolia* is closely related to rice, with a divergence time estimated between 19.7 and 31.0 million years ago.^38^ *Z. latifolia*, a crop species in the Poaceae family closely related to rice, possesses many desirable traits required by modern rice cultivars. It naturally retains numerous excellent characteristics that have been lost in domesticated crops, such as resistance to rice blast disease, robust straw, strong tillering ability, flood tolerance, and cold resistance.^39,40^ In recent years, with the rapid development of plant molecular breeding, researchers have successfully transferred genes of interest from *Z. latifolia* into rice and other model plants, enabling the functional validation and application of high-value genes from this genus.^41^ Low-temperature stress is a common abiotic stress that plants experience under low-temperature conditions, occurring when plants are exposed to temperatures above freezing but below their normal growth temperature range, typically around 0–15°C. Compared with other Poaceae crops, *Z. latifolia* exhibits stronger cold tolerance.^38^ This suggests that it harbors a rich resource of cold-tolerant genes, a trait that provides valuable reference for broadening the genetic base of rice breeding and helps to break through the limitations of genetic resources in traditional rice breeding.^42^

In this study, we conducted a metabolomic analysis of *Z. latifolia* under normal temperature and after 12 hours of cold treatment to elucidate the metabolic mechanisms underlying its cold tolerance. After 12 hours of cold stress treatment, significant changes in the levels of 690 metabolites were observed in *Z. latifolia*, including several compounds known to be involved in abiotic stress responses, such as flavonoids, amino acids and their derivatives, as well as alkaloid metabolites. bZIP-TFs are broadly distributed in plants and play a significant role in plant cold stress responses, as well as in regulating the secondary metabolism of terpenoids, alkaloids, and flavonoids.^43^ However, to date, there have been no reports on the analysis of the full genome bZIP transcription factor family in *Z. latifolia* or on the cold-tolerance genes in this species. This study systematically analyzed the members of the bZIP gene family, identifying a total of 115 bZIP genes in *Z. latifolia*. A detailed investigation was conducted on their basic physicochemical properties, chromosomal locations, gene collinearity, conserved motifs, gene structures, phylogenetic relationships, and cis-acting elements. After screening, the bZIP genes from *Z. latifolia* were aligned with the rice genome using BLAST, yielding four candidate genes potentially associated with cold tolerance. Expression levels of the candidate genes were assessed under various durations of cold treatment to identify key cold-tolerance genes in *Z. latifolia*. This study provides an in-depth investigation of the metabolic changes in *Z. latifolia* under cold stress and the identification and analysis of the bZIP transcription factor family in *Z. latifolia*, aiming to uncover key cold-tolerant genes in this species. These results not only enrich our knowledge of the cold resistance mechanisms in *Z. latifolia* but also have significant implications for the breeding and improvement of crop varieties with enhanced low-temperature tolerance. This knowledge can help to enhance the survival and productivity of cultivated plants in the face of extreme low-temperature environments.

## 2. Materials and Methods

### 2.1. Plant Material and Cold Treatment

*Z. latifolia* seedlings were obtained from the Tonghua Academy of Agricultural Sciences, Meihekou City, Jilin Province, China (42°29′N, 125°46′E), with detailed collection methods described in Yan et al. (2018). After air-drying, sieving, and purification, the seeds were sterilized by soaking in a 0.5% sodium hypochlorite solution (pH adjusted to 5.5 with 6 mol/L hydrochloric acid) and rinsed with sterile deionized water until the pH of the rinse water reached 7.0. An appropriate amount of seeds was placed in an Erlenmeyer flask containing deionized water (seed-to-water ratio of 1:4) and soaked in a 30°C water bath for 5 hours. After soaking, the seeds were transferred to a light incubator for cultivation at 25°C in the dark. When the seedlings reached the five-leaf stage, uniform seedlings were selected and transferred to a growth chamber for cold stress treatment at 4°C (16 h light/8 h dark). The 0 h group was used as the control (A), and cold treatments were applied for 6 h (B), 12 h (C), 24 h (D), and 48 h (E). Leaves from groups A and C were selected for metabolite detection. Three biological replicates were set for each treatment. After each treatment, leaves from individual seedlings were collected, frozen in liquid nitrogen, and stored at − 80°C for RNA extraction.

### 2.2. Metabolomic Analysis

#### 2.2.1. Sample Preparation and Quality Control

Freeze-dried leaf samples (0.1 g) were ground into a fine powder using a mixer mill (MM 400, Retsch) with zirconia beads at 30 hz for 1.5 min to ensure uniform particle size. A pooled quality control (QC) sample was prepared by combining equal volumes of all individual extract supernatants to monitor instrument stability and analytical reproducibility. Extracts were obtained by overnight incubation at 4°C with 0.6 mL of 70% (v/v) aqueous methanol, followed by centrifugation at 10,000×g for 10 min. Reagent blanks (70% methanol without sample) were included to detect potential contamination, and all samples were processed in triplicate (biological replicates) to assess experimental variability.

#### 2.2.2. Instrumental Analysis and Data Acquisition

Before sample analysis, the UPLC-MS/MS system was calibrated using a mass standard solution (Sciex Tune Mix) to ensure mass accuracy (<±5 ppm). QC samples were injected every 10 experimental samples to monitor signal drift and ion suppression, with a total of 6 QC samples included in the analytical batch. Chromatographic separation was performed on a C18 column (1.7 μm, 2.1 × 100 mm) using a gradient of 0.1% formic acid in water (solvent A) and acetonitrile (solvent B): 5–10% B (0–2 min), 10–40% B (2–12 min), 40–95% B (12–14 min), 95% B (14–16 min), 95–5% B (16–16.1 min), and 5% B (16.1–20 min) at a flow rate of 0.3 mL/min. The ESI source operated in both positive and negative ion modes with the following parameters: ion spray voltage, 5500 V (positive) and −4500 V (negative); curtain gas, 30 psi; collision gas, medium; ion source temperature, 550°C.

#### 2.2.3. Data Preprocessing and Differential Metabolite Screening

Raw data were processed using Analyst software (v1.6.3, AB Sciex) for peak extraction, alignment, and integration. Peak intensities were normalized by total ion current (TIC) to correct for variations in injection volume and ionization efficiency. Missing values in the metabolite matrix were imputed using the k-nearest neighbors (KNN) algorithm for samples with ≥ 50% valid data within groups. Differential metabolites were identified using the criteria of variable importance in the projection (VIP) ≥ 1 (from orthogonal partial least squares discriminant analysis, OPLS-DA) and absolute Log2(fold change) ≥ 1, with significance further validated by Student’s t-test (*p* < .05). Principal component analysis (PCA) of QC samples was performed to evaluate data quality, showing tight clustering and low inter-batch variability. Pathway enrichment was conducted using the KEGG database, and only pathways with a corrected P-value <.05 were considered significant.

### 2.3. Data Analysis

Principal component analysis (PCA) and hierarchical clustering were performed using the procomp function and COMPLEXHEATMAP in R software (www.r-project.org.), respectively, to compare the metabolite profiles among different time points in the leaves of *Z. latifolia*. The transformed and Z-score normalized metabolite accumulation data were used for PCA analysis and hierarchical clustering. Enrichment analysis was performed based on the hypergeometric test. Pathway enrichment analysis was performed based on the KEGG database.

### 2.4. Identification of bZIP Transcription Factor Families in Z. Latifolia

Rice bZIP protein sequences were downloaded from PlantTFDB (3.0) (http://planttfdb.gao-lab.org). The whole-genome data of *Z. latifolia* (genomic sequence, coding sequence, and protein sequence) were obtained from Genome Warehouse (https://ngdc.cncb.ac.cn/gwh/Assembly/22880/show). Using rice bZIP sequences as query sequences, candidate bZIP genes in the Z. latifolia genome were first identified by BLAST. Then, the hidden Markov model (HMM) of the bZIP family genes was downloaded from the PFAM database (http://pfam.xfam.org.), and the presence of bZIP conserved domains was verified using PFAM, NCBI Conserved Domain Database (http://www.ncbi.nlm.nih.gov/Structure/cdd/wrpsb.cgi.), and SMART database (http://smart.embl-heidelberg.de/).

### 2.5. Phylogenetic Analysis of bZIP Transcription Factor Family Genes Phylogenetic Analysis

Chromosomal location information of all bZIP genes in the *Z. latifolia* genome was extracted, and the chromosomal localization map of the ZlbZIP family genes was drawn using the online tool MapGene2Chromosome v2 (http://mg2c.iask.in/mg2c_v2.0). The neighbor-joining (NJ) method in MEGA7 software was used to construct a phylogenetic tree of protein sequences from *Z. latifolia* and rice, with bootstrap parameters set to 1000 and other parameters using system defaults.

### 2.6. Analysis of Motifs, Gene Structures, and Conserved Structural Domains

Exon, intron, and untranslated region positions of each *Z. latifolia* bZIP gene were obtained from the genome. Conserved motifs in *Z. latifolia* bZIP genes were identified using the MEME server (https://meme-suite.org.) with parameters set as follows: maximum number of motifs = 10, minimum motif width = 6, maximum motif width = 100.^44^ Domain analysis was performed using the NCBI Conserved Domain Database (CDD) to determine the type and position of all domains in protein sequences. The exon/intron structure of bZIP genes and the conserved motifs and domains of bZIP proteins were visualized using TBtools software.^45^

### 2.7. Gene Duplication and Collinearity Analysis of the bZIP Transcription Factor Family

The Multiple Collinearity Scan Toolkit (MCScanX) software^46^ was used to identify homologous gene pairs and collinear relationships in the *Z. latifolia* bZIP family. Additionally, the Ka/Ks Calculator 2.0 was used to calculate the ratio of non-synonymous substitution rate (Ka) to synonymous substitution rate (Ks) for two protein-coding genes as an indicator of nucleic acid molecular evolution to determine whether protein-coding genes were under selective pressure. To predict the functions of bZIP family genes, collinear relationships between *Z. latifolia* and rice bZIP family genes were studied using MCScanX software. The duplication and homologous genetic relationships of bZIP family genes between *Z. latifolia* and rice were visualized using advanced circus plots in TBtools.

### 2.8. Analysis of Promoters for the bZIP Transcription Factor Family Genes

Nucleotide sequences of 1500 bp upstream of the start codon of each bZIP gene were extracted from the *Z. latifolia* genome and submitted to the PlantCARE database (https://bioinformatics.psb.ugent.be/webtools/plantcare/html/) for cis-regulatory element prediction.^47^ Predicted cis-regulatory elements were classified according to their regulatory functions, and cis-regulatory elements related to cold stress were visualized using TBtools.

### 2.9. Identification of Cold Tolerance Genes in Z. Latifolia

Cold tolerance-related genes in O. sativa were obtained by querying gene names on the *Z. latifolia* Data Centre website (https://www.ricedata.cn/). Cold tolerance genes in *Z. latifolia* were identified by comparing similar genes in O. sativa with the *Z. latifolia* genome sequences in this study. The e-value of sequence alignment results was set to < 1 × 10 − 10. MCScanX was used for collinearity analysis of candidate genes.

### 2.10. RNA Extraction and Quantitative Real-Time PCR Analysis

RNA was extracted from leaf samples of the control group (A, 0 h) and treatment groups (B, 6 h; C, 12 h; D, 24 h; E, 48 h) using the TaKaRa MiniBEST Plant RNA Extraction Kit for gene expression pattern analysis. cDNA was synthesized using a reverse transcription kit (TaKaRa, Dalian, China) according to the manufacturer’s instructions. Real-time quantitative PCR (qRT-PCR) was performed using the SYBR Premix Ex Taq™ Kit (TaKaRa) on a Bio-Rad iQ1 Real-Time PCR system (Bio-Rad). *UBQ5* was used as the internal reference gene to normalize target gene expression. Data were calculated using the 2^−ΔΔCt^ method, with the final value averaged from triplicate reactions. The Ct value of CsActin was used to normalize the Ct value of each gene. Experiments were repeated at least three times. Primer sequences used in this study are provided in Supplemental Table S6.

### 2.11. Statistical Analysis

All experiments performed in this study were repeated at least twice with three biological replicates. The mean±SD (standard deviation) were calculated and processed using one-way analysis of variance (ANOVA) in SPSS (IBM, NY, USA) and t-tests, with *p < .05, **p < .01 and ***p < .001 indicating statistically significant differences.

## 3. Results

### 3.1. Metabolomic Profiles of Z. Latifolia in Response to Cold Stress

**Figure 1.**
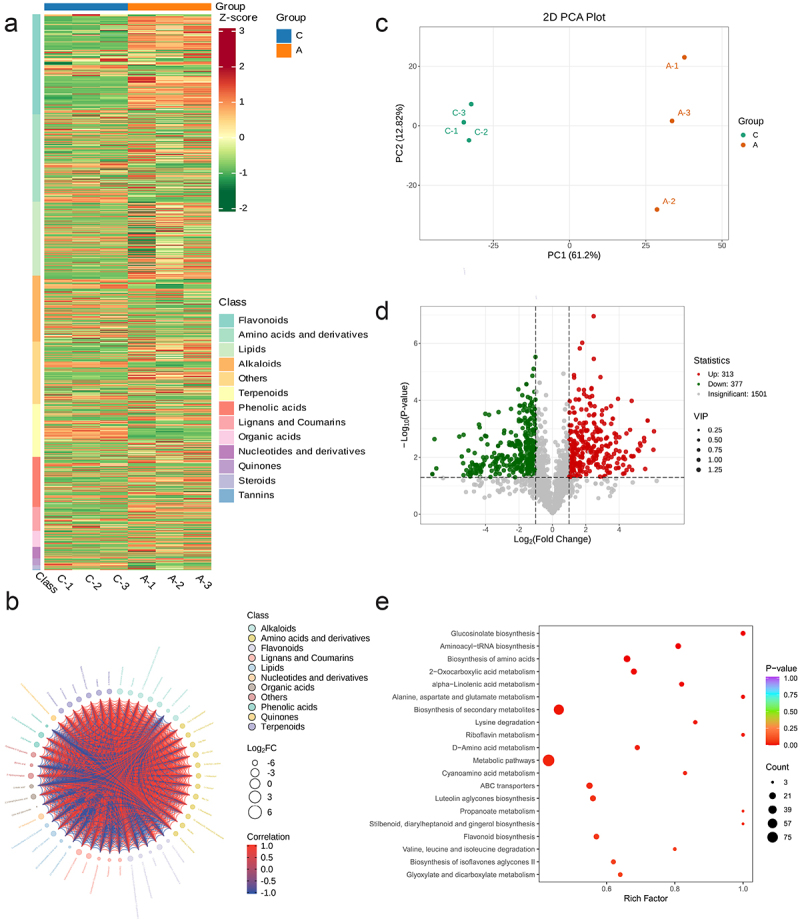
Analysis of metabolic variation in *Zizania latifolia* (*Z.Latifolia*) under cold treatment. (a) Hierarchically clustered heatmap of the 810 metabolites from *Z.Latifolia* after cold treatment. The false color scale is depicted on the right side of the image. (b) Association and changes of differential metabolites between control a (0 h) and treatment C (12 h). (c) Principal component analysis (PCA) of all metabolites generated in different time points. (d) volcano plot, the color of each scatter point indicates the final screening results: red-significantly up regulated phenolic metabolites, green-significantly down regulated phenolic metabolites, and gray-phenolic metabolites whose levels did not significantly differ between A and C. (e) KEGG enriched pathways of differential metabolites. The vertical axis represents the names of pathways, whereas the horizontal axis represents the rich factor of each pathway. The color of each point in this figure represents its p-value (purple to red indicates low to high significant enrichment), and the point size represents the number of differential metabolites annotated to their corresponding pathways.

To investigate the differential changes in metabolites of *Z. latifolia* under cold stress, leaf samples from the control group (A) and the 4°C cold-treated group (C) for 12 hours were analyzed using a widely targeted metabolomics approach with a UPLC-ESI-MS/MS system. A total of 810 metabolites were identified, belonging to 13 major categories including 146 flavonoids, 127 amino acids and derivatives, and 96 alkaloids (Figure 1(a), Table S1). Flavonoids such as cyanidin-3-O-glucoside and quercetin-3-O-rutinoside, amino acid derivatives like proline and glycine betaine, and alkaloids such as 5-hydroxytryptamine and tyramine significantly accumulated after cold treatment, becoming core components of differential metabolism.^10,48,49^

Differential metabolite analysis showed significant differentiation in metabolite profiles between the experimental and control groups after 12 hours of cold treatment. Principal component analysis (PCA) revealed tight clustering within each sample group, with a clear separation between the control group (A) and the treatment group (C) (Figure 1(c)), consistent with hierarchical clustering results, indicating a systematic impact of cold stress on the metabolome. According to the criteria of VIP > 1 and fold change ≥ 2 (upregulated)/≤0.5 (downregulated), 690 differentially accumulated metabolites (DAMs) were screened, including 313 upregulated and 377 downregulated metabolites (Figure 1(d)). KEGG enrichment analysis showed that 90 pathways were enriched with differential metabolites, with notable activation of flavonoid biosynthesis (e.g., flavonols, isoflavones), amino acid metabolism (phenylalanine, tyrosine), and alkaloid synthesis (phenolic amines, indole alkaloids) pathways, which are closely related to antioxidant defense, osmotic adjustment, and signal transduction functions under cold stress.^48,50,51^

Notably, flavonoids scavenge cold-induced superoxide anions (O₂^−^) and hydrogen peroxide (H₂O₂) through hydroxylated structures. For example, quercetin inhibits the production of malondialdehyde (MDA), a product of membrane lipid peroxidation, and maintains membrane liquid crystalline fluidity.^10,11^ Amino acid derivatives like proline act as osmotic regulators, reducing the freezing point by increasing cytosolic concentration, and their imino groups form hydrogen bonds with water molecules to prevent cytoplasmic dehydration, which has been confirmed to directly correlate with seedling survival rate in japonica rice under cold stress.^49,50^ Alkaloids such as tryptamine may participate in cold signal transduction by regulating calcium channels or activating antioxidant enzyme systems (e.g., SOD), with similar functions reported in maize cold stress responses.^43,48^ The directional accumulation of these metabolites collectively forms a chemical barrier for *Z. latifolia* to resist cold stress, reflecting the coordinated regulation mechanism of primary and secondary metabolism in stress responses. The synergistic effect of these metabolites’ directional accumulation and transcriptional regulatory networks reveals the unique strategy of *Z. latifolia* in coping with cold stress, not only providing new insights into the molecular basis of its cold tolerance but also laying an important foundation for further mining of excellent gene resources and breeding of cold-resistant crop varieties.

Furthermore, the principal component analysis (PCA) results shown in Figure 1(c) indicate that among the selected samples, the distances between individual samples within each group are relatively close, while the differences between different groups are quite pronounced. A clear trend of clustering was observed between control group A and treatment group C, which is consistent with the results of hierarchical cluster analysis. By comparing the metabolomics data with the criteria of VIP (Variable Importance in Projection) values greater than 1 and fold changes greater than 2 (upregulated) or less than 0.5 (downregulated), a total of 690 differentially accumulated metabolites (DAMs) were identified, and a volcano plot was generated. From Figure 1(d), it can be observed that when comparing the control group A with the group C after 12 hours of cold treatment, a total of 313 metabolites showed significant upregulation, while 377 metabolites were notably downregulated. Furthermore, to gain a deeper understanding of the metabolic changes during the cold stress response, enrichment analysis was conducted on the differential metabolites between the two groups using the Kyoto Encyclopedia of Genes and Genomes (KEGG) analysis, yielding the enrichment results of metabolic pathways (Figure 1(e), Table S2). In the comparison between the control group A and the treatment group C, a total of 90 pathways were enriched with differential metabolites. Figures reveal that, following cold treatment, metabolites involved in primary and secondary metabolic biosynthesis pathways accumulated significantly compared to the control group. Among them, flavonoids (flavones, isoflavonoids, dihydroflavonols), amino acids and their derivatives (phenylalanine, tyrosine, etc.), and alkaloids (alkaloids, phenols, and indole alkaloids) showed very significant differences in the accumulation of metabolites under cold stress. In summary, it is clear that flavonoids, amino acids and their derivatives, and alkaloids play a crucial role in the metabolic and secondary metabolic biosynthetic pathways of *Z. latifolia*, which are essential for the plant’s defense against cold stress.

### 3.2. Identification of ZlbZIP Family TFs and Chromosome Localisation

**Figure 2.**
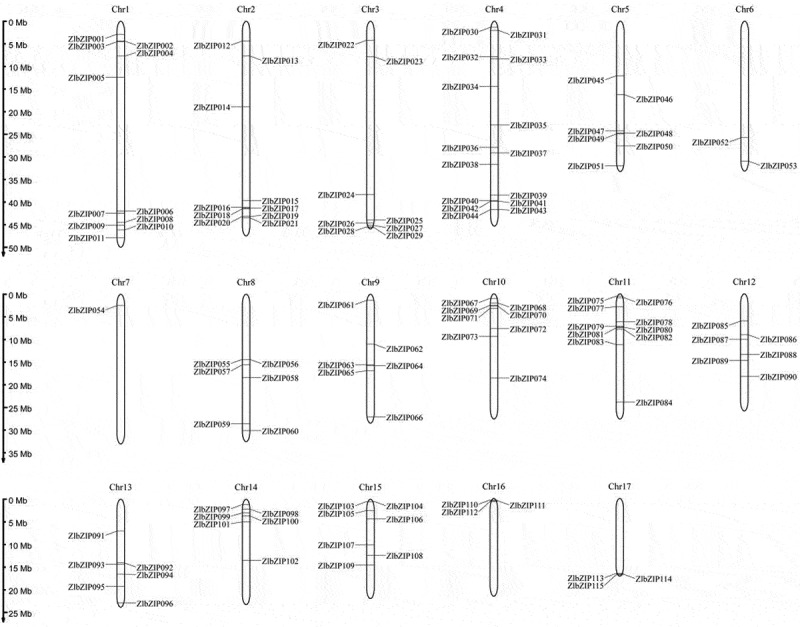
Chromosomal locations of bZIP genes in *Z.Latifolia*. The gene IDs of the ZlbZIP family are listed in table S3.

Based on BLAST and HMM analyses of the PlanTFDB (http://planttfdb.gao-lab.org) genome database, pseudogenes, premature termination codon genes, and genes without complete MADS domains were removed. Finally, 115 ZlbZIP genes were identified in the *Z. latifolia* genome and named ZlbZIP001–ZlbZIP115 in order of their chromosomal positions (Figure 2, Table S3). Using MapInspect software, the 115 ZlbZIP genes were mapped to 17 chromosomes of *Z. latifolia*, with significant gene clusters (gene spacing < 50 kb) on chromosomes 5 (14 genes), 3 (12 genes), and 1 (11 genes). Comparison with the Gramene QTLdb v2.0 database revealed that 18 ZlbZIP genes were distributed in reported cold-resistant QTL intervals, mainly concentrated in the following regions: the qCTS1.1 interval (3.2–4.5 Mb) on chromosome 1 contained *ZlbZIP005* and *ZlbZIP021*, completely overlapping with the previously mapped seedling low-temperature survival rate QTL. The promoter region of this interval was enriched with G-box elements closely related to cold response.

The qLTG5.2 homologous interval (12.8–14.2 Mb) on chromosome 5 was enriched with 7 genes including *ZlbZIP075*, corresponding to the rice low-temperature germination rate QTL region, which showed linkage disequilibrium under cold stress. Cross-species collinearity analysis showed that *Z. latifolia ZlbZIP005* shared 89% homology with the rice cold-resistant gene OsbZIP71 (located in the cold-sensitive QTL qCTB1 on rice chromosome 1), and both promoter regions contained high-density G-box elements; *ZlbZIP038* formed an orthologous pair with the maize cold-resistant QTL (qCT3.1) core gene ZmbZIP4, with sequence similarity supporting their conserved functions in the flavonoid metabolism pathway.

The chromosomal localization characteristics of these ZlbZIP genes functionally correspond to the results of differential metabolite pathway enrichment: genes like *ZlbZIP005* and *ZlbZIP075* located in cold-resistant QTL intervals may directly participate in cold stress responses in antioxidant and osmotic adjustment pathways by regulating key enzyme genes such as flavonoid synthase (CHS/FLS) and proline synthase (P5CS), reflecting a synergistic mechanism of “genetic mapping-gene expression-metabolic regulation.” Cross-species collinearity analysis further indicates that *ZlbZIP* genes and their regulated metabolic pathways in cold-resistant QTL regions are conserved in gramineous crops, providing multi-dimensional evidence for analyze the molecular basis of cold tolerance in *Z. latifolia*.

### 3.3. Phylogenetic Analysis of ZlbZIP Genes

**Figure 3.**
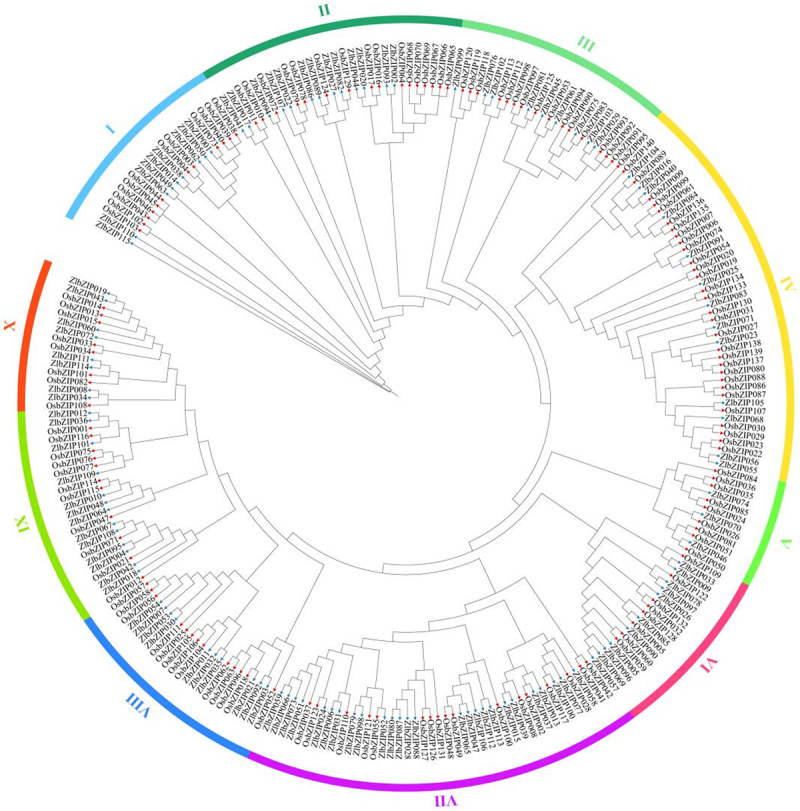
Phylogenetic analysis of bZIP transcription factors in *Z. latifolia* (zl), and *oryza sativa* (os). The blue arrow and red arrow represent *Z. latifolia* protein and *oryza* sativa protein, respectively. The different colors of the outer circle are used to denote bZIP groups and subgroups.

To further investigate the phylogenetic relationships and functional divergence of the ZlbZIP transcription factor family, a phylogenetic tree was constructed using 115 *Z. latifolia* ZlbZIP and 140 rice OsbZIP protein sequences. The results (Figure 3) revealed that, similar to rice, the 115 ZlbZIP members in *Z. latifolia* were also grouped into 10 subfamilies. However, significant differences in subfamily sizes were observed: Subfamily Ⅶ contained the largest number of bZIP genes in *Z. latifolia* (26 members) compared to 17 in rice, while Subfamily Ⅳ had the highest number in rice (31 members) but only 13 in *Z. latifolia*. Subfamily Ⅴ had the fewest ZlbZIP genes (3 members) in *Z. latifolia*, whereas Subfamilies Ⅵ and X had the fewest OsbZIP genes (8 members each) in rice.

To reveal the evolutionary relationships and functional differentiation of the ZlbZIP family, a phylogenetic tree with Bootstrap = 1000 was constructed. Both species were divided into 10 subfamilies (Figure 3), but notable variations in subfamily membership were evident: Subfamily Ⅶ in *Z. latifolia* had 26 members, significantly more than rice’s 17; rice Subfamily Ⅳ, with 31 members, was approximately 2.4 times larger than that of *Z. latifolia* (13 members), indicating lineage-specific expansions and contractions during evolution. Comparison with cold-tolerant model species (Arabidopsis, rice) revealed both evolutionary conservation and species-specific adaptations in gene structure, conserved motifs, and cis-acting elements:

Conserved Gene Structure and Functional Correlation

**Figure 4.**
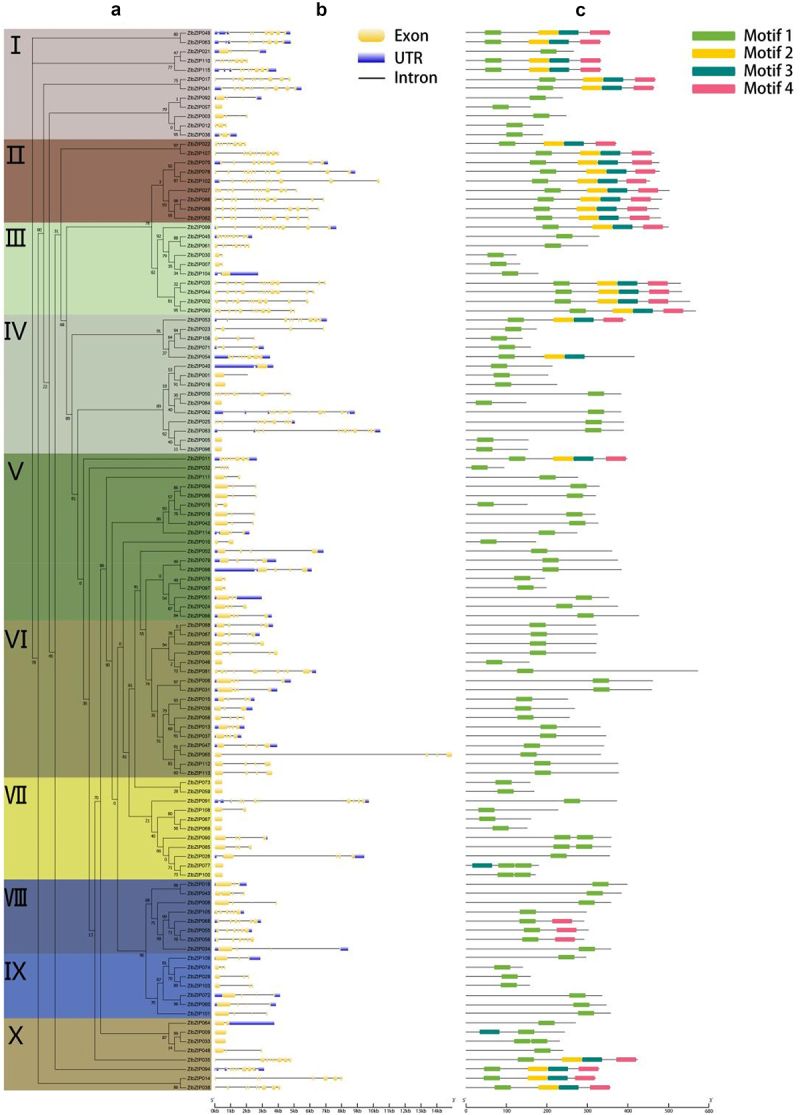
Conserved motif analysis of *Z. latifolia* basic-region leucine zipper (bZIP) transcription factors (TFs): (a) phylogenetic tree, (b) gene structure, and (c) conserved motifs. In part (a), the different background colors represent different subfamilies. In part (b), the yellow boxes and black lines represent the exons and introns, respectively. In part (c), different colors represent different conserved motifs.

The number of exons in *Z. latifolia* ZlbZIP genes ranged from 1 to 15, with 61.74% containing 1–4 exons (Figure 4(b)), highly similar to rice OsbZIP genes (1–13 exons, 58% with 1–4 exons)^52^ but significantly more than Arabidopsis AtbZIP genes (mostly 1–3 exons).^27^ In the cold-related Subfamily Ⅶ, candidate genes such as *ZlbZIP005* and *ZlbZIP075* all had 2–3 exons, consistent with the structure of rice cold-tolerant genes *LIP19* (2 exons) and *OsbZIP7*1 (3 exons), suggesting that conserved exon numbers may be associated with stable cold-responsive regulation. Relatively simple gene structures (fewer introns) may facilitate rapid transcriptional activation under cold stress, functionally aligning with the report that “genes with fewer introns have lower expression levels in plants.”^53^

(1) Functional Differentiation of Conserved Motifs

Four conserved motifs (Motif 1–4) were identified in *Z. latifolia* ZlbZIP proteins, with Motif 1 (core sequence RLRKLLSP) shared by all subfamilies, corresponding to the basic region and leucine zipper start site of the bZIP domain (Figure 4(c)). This showed high homology with the key functional domains of Arabidopsis *AtbZIP1* (89% similarity) and rice *OsbZIP73* (92% similarity),^27,52^ indicating the core role of this motif in bZIP family evolution. Notably, cold-responsive candidate genes *ZlbZIP005* and *ZlbZIP084* possessed an additional Motif 3 (rich in serine phosphorylation sites) downstream of Motif 1, similar to the phosphorylation regulatory sites of the Arabidopsis cold-induced gene *AtbZIP28*.^21^ This suggests they may enhance transcription factor activity through MAPK cascade-mediated phosphorylation, playing a key role in cold signal transduction.

(2) Enrichment Differences in Cis-Acting Elements and Regulatory Potential

Promoter cis-acting element analysis showed that 92.2% (106/115) of *Z. latifolia ZlbZIP* genes were highly enriched in G-box (CACGTG), a cold-responsive core element, significantly higher than in rice (65.7%, 92/140) and Arabidopsis (90.7%, 68/75).^27,52^ As a core regulatory element for cold response, G-box specifically binds bZIP proteins to activate downstream genes (e.g., COR15A).^18^ For example, the rice cold-tolerant gene OsbZIP71 has 3 G-boxes in its promoter, while the homologous gene *ZlbZIP075* in *Z. latifolia* has 5 G-boxes in its promoter region, suggesting stronger cold-inducible potential. Additionally, the enrichment of methyl jasmonate response elements (CGTCA-motif/TGACG-motif) in cold-related subfamilies further indicates that *ZlbZIP* genes may construct complex regulatory networks by integrating hormone signals with cold stress responses.

(3) Evolutionary Characteristics and Functional Implications

The *Z. latifolia* bZIP family showed high phylogenetic homology with rice but formed differentiated cold response mechanisms through adaptive adjustments in subfamily membership (e.g., Subfamily Ⅶ expansion), acquisition of specific motifs (e.g., Motif 3), and cis-element enrichment. These characteristics not only provide a structural basis for candidate gene functional studies but also offer evolutionary biological evidence for cross-species cold-tolerant gene mining and utilization.

### 3.4. Analysis of Gene Structure and Conserved Motifs of the ZlbZIP Gene Family

Phylogenetic analysis of ZlbZIP gene sequences revealed clustering into 10 subfamilies (Figure 4(a)). Comparison of intron/exon structures showed that the number of exons in *ZlbZIP* genes ranged from 1 (e.g., *ZlbZIP005, ZlbZIP009*) to 15 (*ZlbZIP062*), with most genes (61.74%) containing 1–4 exons and lengths exceeding 1.6 kb (Figure 4(b)). Intron numbers varied significantly, with *ZlbZIP062* having the most (14 introns). Notably, 15 genes contained two introns, 6 had one intron, and 14 lacked introns entirely, consistent with the observation that genes with few or no introns often exhibit lower expression levels in plants.^53^

To further explore the functional diversity of ZlbZIP proteins, MEME software was used to predict conserved motifs. Four motifs (Motif 1–4) were identified, with Motif 1 being the most conserved and present in all subfamilies, indicating its critical role in the ZlbZIP family (Figure 4(c)). Closely related ZlbZIP proteins within phylogenetic clades (e.g., *ZlbZIP088, ZlbZIP087, ZlbZIP028, ZlbZIP080, ZlbZIP046*) shared identical or similar motif compositions, validating the phylogenetic classification. These stable domain architectures are key determinants of phylogenetic relationships within protein families.

**Figure 5.**
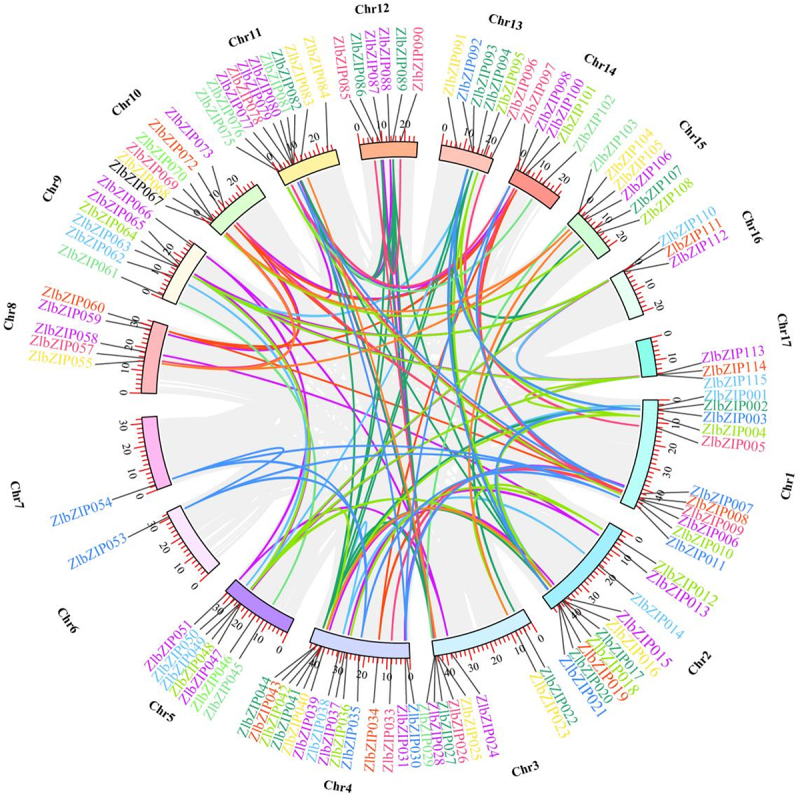
Collinearity analysis of *bZIP* genes in *Z. latifolia*. Gene IDs with the same color represent genes of the same *bZIP* subfamily. Gene subfamily color reference figure 2.

Gene duplication analysis revealed that segmental duplication was the primary expansion mechanism for the ZlbZIP family, with 121 homologous gene pairs identified by MCScanX: only 2 pairs were tandem duplicates, while the remaining 119 were interchromosomal segmental duplicates (Figure 5). Ka/Ks ratio analysis indicated that 78.5% of gene pairs were under purifying selection (Ka/Ks < 1, mean 0.62), maintaining functional conservation, whereas 15.7% (e.g., *ZlbZIP005-ZlbZIP021*, Ka/Ks = 1.23) experienced positive selection, suggesting functional divergence post-duplication. These duplication events drove functional diversification through multiple mechanisms:

Differential cis-element enrichment led to subfunctionalization, as exemplified by *ZlbZIP005* (5 cold-responsive elements in promoter) vs. its segmental duplicate *ZlbZIP021* (2 elements), likely conferring distinct cold-inducible expression kinetics.

Domain innovation occurred through coding region mutations, such as *ZlbZIP075* acquiring an EAR repression domain (LxLxL motif) absent in its homolog *ZlbZIP100*, potentially enabling roles in ABA signaling repression versus metabolic pathway activation.

Promoter targeting divergence in tandem duplicates (e.g., *ZlbZIP038-ZlbZIP021*) allowed coordinated regulation of flavonoid metabolism enzymes (e.g., CHS and FLS), enhancing pathway efficiency.

This segmental duplication-driven expansion strategy enabled the ZlbZIP family to maintain core cold tolerance functions (e.g., membrane protection, osmotic adjustment) through purifying selection while diversifying regulatory networks via positive selection. These mechanisms provide genetic flexibility for *Z. latifolia* to adapt to cold stress and offer insights into the evolution-function relationship of plant stress-responsive gene families.

### 3.5. Analysis of GO Classification Results for the ZlbZIP Gene Family

**Figure 6.**
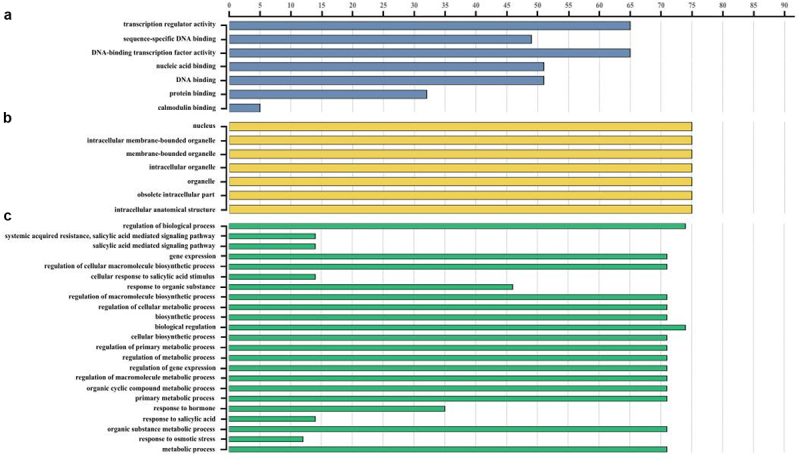
Gene ontology (GO) annotation of ZlbZIP proteins. The annotation was performed on three categories, (A) molecular function, (B) cellular componentand (C) biological process.

Beyond the conserved bZIP domain, the highly divergent sequences suggest that bZIP proteins are involved in diverse biological processes. Given the functional characterization of multiple bZIP proteins in rice, GO annotation was performed for *Z. latifolia* bZIP proteins using rice as a reference species. The results (Figure 6, Table S4) showed that most ZlbZIP proteins were associated with “DNA-binding transcription factor activity” and DNA/nucleic acid binding. Additionally, 32 proteins were involved in protein binding, and five proteins regulated calmodulin binding.

In terms of cellular components, 75 ZlbZIP proteins were predicted to localize to the nucleus and organelles, reflecting their multifunctionality across biological processes. Biological process analysis revealed that “biological regulation” and “regulation of cellular metabolic processes” were the most highly represented categories. ZlbZIP proteins also responded to “regulation of primary metabolic processes” and “regulation of macromolecule metabolic processes.” Furthermore, 12–46 ZlbZIP proteins were involved in responses to organic substances, hormonal stimuli (e.g., jasmonate, salicylic acid), and osmotic stress.

Collectively, these GO annotations indicate that ZlbZIP proteins play versatile roles in biosynthesis and metabolism to mediate responses to both abiotic and biotic stresses.

### 3.6. Analysis of Cis-Acting Elements in the Promoter Region of ZlbZIP Genes

Analysis of promoter cis-acting elements is crucial for understanding transcriptional regulation. To investigate the expression and regulation of ZlbZIP transcription factors, we analyzed their promoter regions using the PlantCARE database. Results revealed enrichment of multiple abiotic stress-responsive elements (Figure S1), including the cold-responsive G-box (CACGTG, 92.2%), methyl jasmonate-responsive elements (CGTCA-motif/TGACG-motif, 68.7% and 59.1%, respectively), and low-temperature-responsive elements (LTR, 51.3%). The G-box, a core binding site for bZIP TFs, was present in 106 ZlbZIP promoters, significantly higher than in rice (65.7%) and Arabidopsis (90.7%).^27,52^ For example, the rice cold-tolerant gene OsbZIP71 contains 3 G-boxes, while its homolog *ZlbZIP075* in *Z. latifolia* harbors 5, suggesting stronger cold-inducible potential.^18,33^

Comparison with cold-responsive genes from other species showed that G-box enrichment is conserved in cold-inducible genes such as Arabidopsis COR15A and rice LIP19.^18,51^ Jasmonate-responsive elements (CGTCA/TGACG-motifs), also reported in rice cold-related subfamilies, suggest that *ZlbZIP* genes may integrate hormonal signaling (e.g., jasmonate pathway) with cold stress responses to form complex regulatory networks.^32,35^ Notably, the enrichment of LTR elements (51.3%) in *Z. latifolia* exceeded Arabidopsis (42.7%) but was lower than rice (63.2%), reflecting evolutionary divergence in cold signal perception modules.^52,54^

Differential cis-element enrichment underscores functional specificity: the high proportion of G-boxes ensures efficient binding of bZIP proteins to downstream targets (e.g., flavonoid synthase CHS, osmolyte synthase P5CS), while jasmonate-responsive elements suggest involvement in stress-growth crosstalk.^43,50^ Cross-species comparisons highlight conservation of core cold-responsive elements (e.g., G-box) in gramineous crops, with species-specific variations in element density and combinations, providing evolutionary evidence for the transcriptional regulation of cold tolerance in *Z. latifolia*

### 3.7. Amino Acid Sequence Alignment of ZlbZIP Homologous Genes

**Figure 7.**
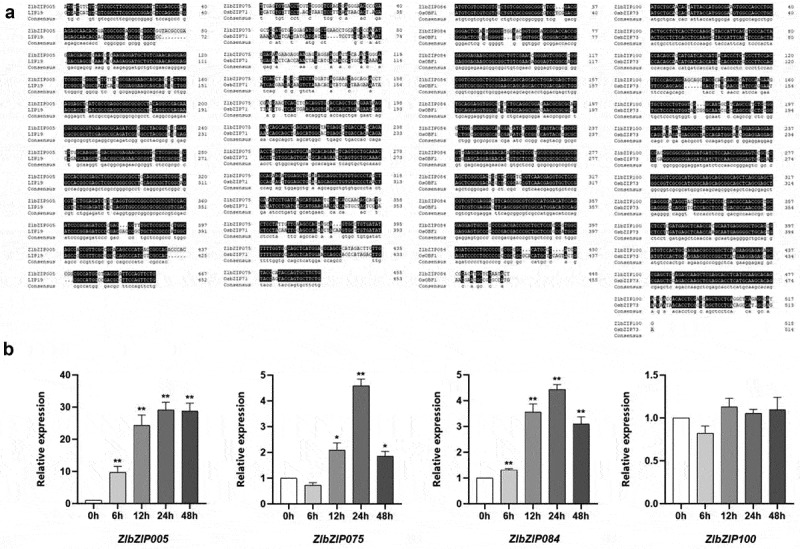
Alignment of amino acid sequences of ZlbZIP and expression analysis of candidate genes in different materials. (a) *ZlbZIP005, ZlbZIP075, ZlbZIP084, ZlbZIP10*0 were derived from *Z. latifolia*, *OsbZIP71, LIP19, OsOBF1, OsbZIP73* were derived from *Oryza sativa*. (b) Differential expression plots of candidate genes under different low temperature stress treatment time.

In previous studies, it was found that the *Z. latifolia* genome shares significant collinearity with the rice genome.^38^ To our knowledge, 193 cold-tolerance related genes have been identified in rice, among which there are six bZIP transcription factors: *Oreb1, Risbz5, Lip19, OsOBF1, OsbZIP71, and OsbZIP73* (Table S5). Analysis of collinearity and gene homology between the *Z. latifolia* and rice genomes indicates that four *ZlbZIPs* from *Z. latifolia* are homologous to known cold-tolerance genes in rice. Specifically, *ZlbZIP005* (Zla01G014100) on chromosome 1 is homologous to *LIP19* (LOC_Os05g03860) in rice. *ZlbZIP075* (Zla11G000470) on chromosome 10 is homologous to *OsbZIP71* (LOC_Os09g13570) in rice. *ZlbZIP084* (Zla11G015400) on chromosome 11 is homologous to *OsOBF1* (LOC_Os12g37410) in rice; ZlbZIP100 (Zla14G005100) on chromosome 13 is homologous to *OsbZIP73* (LOC_Os09g29820) in rice. Figure 7(a) displays the results of the amino acid alignment between *Z. latifolia* and rice homologous genes, with background color indicating the degree of sequence similarity, where black represents 100% identity. The amino acid sequences of the homologous genes from *Z. latifolia* and rice exhibit a high degree of similarity. It is known that these homologous genes in rice are members of the bZIP transcription factor family and play a crucial regulatory role in plant cold tolerance and response to low-temperature stress. Therefore, it is inferred that *ZlbZIP005, ZlbZIP075, ZlbZIP084, and ZlbZIP100* are important transcription factors in *Z. latifolia*, participating in the regulation of the plant’s response to cold stress. It is noteworthy that the sequence alignment of cold-tolerance related genes between *Z. latifolia* and rice aids in identifying potential functional polymorphisms and specific genomic editing candidate sites in *Z. latifolia*.

### 3.8. Expression of Cold Tolerance Genes and Validation by qRT-PCR

To validate the functions of candidate genes in cold stress responses, qRT-PCR analysis was conducted on *Z. latifolia* leaves subjected to 4°C treatment for 0 h, 6 h, 12 h, 24 h, and 48 h. UBQ5 was used as the internal reference gene to normalize expression levels. One-way analysis of variance (ANOVA) with Dunnett’s test was employed for intergroup significance analysis, where *p* < .05, *p* < .01, and *p < .001 indicated significant, highly significant, and extremely significant differences, respectively.

The results (Figure 7(b)) showed that *ZlbZIP005* exhibited a unimodal expression pattern, increasing initially and then decreasing with prolonged cold treatment: expression was upregulated 9.67-fold at 6 h compared to the control (*p* < .01), peaked at 24 h (29.17-fold, *p < .001), and decreased to 18.52-fold at 48 h while remaining significantly higher than the control (*p* < .05). *ZlbZIP075* showed transient downregulation at 6 h (*p* < .05) followed by a gradual increase, with a 4.13-fold upregulation at 24 h (*p* < .01) and sustained expression at 3.21-fold at 48 h (*p* < .05). *ZlbZIP084* displayed a mild 1.31-fold upregulation at 6 h (*p* < .05), reached a peak of 4.10-fold at 24 h (*p* < .01), and decreased to 2.89-fold at 48 h while still being significantly higher than the control (*p* < .05). All three genes reached their expression peaks at 24 h, demonstrating significant temporal induction characteristics.

In contrast, *ZlbZIP100* showed no significant expression differences across all treatment time points compared to the control (*p* > .05), suggesting it does not directly participate in cold stress responses. Collectively, the differential expression patterns of *ZlbZIP005, ZlbZIP075*, and *ZlbZIP084*—particularly their significant upregulation at 24 h – imply that these three genes are involved in *Z. latifolia*’s cold stress response through temporal regulation, providing critical targets for subsequent functional validation of cold-tolerant genes.

## 4. Discussion

### 4.1. Phylogenetic Characteristics of the Z. Latifolia bZIP Transcription Factor Family and Differentiation of Cold-Associated Subfamilies

Phylogenetic analysis is a key approach to decipher functional divergence within gene families. A phylogenetic tree constructed based on 115 *Z. latifolia* ZlbZIP and 140 rice OsbZIP proteins revealed that both families were divided into 10 subfamilies, but with striking differences in subfamily sizes: Subfamily Ⅶ in *Z. latifolia* emerged as the largest with 26 members, representing a 47% expansion compared to rice’s 17 members, while rice Subfamily Ⅳ (31 members) was 2.4-fold larger than its *Z. latifolia* counterpart (13 members).^27,52^ Such lineage-specific expansion and contraction reflect functional adaptations during species evolution to environmental pressures. Notably, Subfamily Ⅶ in rice is enriched with cold-related genes (e.g., *OsbZIP71, OsbZIP73*),^33,34^ suggesting that its expansion in *Z. latifolia* may provide a transcriptional regulatory basis for stronger cold tolerance.

Compared with model species like Arabidopsis and maize, the *Z. latifolia* ZlbZIP family exhibited a mix of conservation and specificity in gene structure: 61.74% of genes contained 1–4 exons, matching the structure of rice cold genes LIP19 (2 exons) and *OsbZIP71* (3 exons) but exceeding Arabidopsis AtbZIP genes (mostly 1–3 exons).^52,53^ This compact gene structure (fewer introns) likely facilitates rapid transcriptional activation under cold stress, functionally aligning with reports that “genes with fewer introns exhibit lower basal expression but faster inducibility.”^53^

Conserved motif analysis showed that all subfamilies harbored the core Motif 1 (RLRKLLSP), corresponding to the basic region and leucine zipper initiation site of the bZIP domain, with homology exceeding 89% to key functional domains in Arabidopsis *AtbZIP1* and rice *OsbZIP73*,^27,52^ underscoring its core role in bZIP family evolution. Notably, cold candidate genes *ZlbZIP005* and *ZlbZIP084* possessed an additional Motif 3 (rich in serine phosphorylation sites) downstream of Motif 1, analogous to the phosphorylation regulatory sites in the Arabidopsis cold-induced gene *AtbZIP28*.^21^ This suggests they may enhance transcription factor activity via MAPK cascade-mediated phosphorylation, playing pivotal roles in cold signal transduction.

### 4.2. Structural and Functional Relationships of Key Cold-Tolerant Candidate Genes

Through cross-species collinearity analysis and expression validation, three key cold-tolerant candidate genes (*ZlbZIP005, ZlbZIP075, ZlbZIP084*) were screened, whose structural characteristics and cold response patterns reveal distinct functional differentiations:

*ZlbZIP005* (orthologous to rice *LIP19*): Located in the qCTS1.1 interval on chromosome 1 (seedling low-temperature survival rate QTL), this gene has 2 exons, and its promoter region is enriched with five G-box elements (more dense than the three G-boxes in the rice homolog *LIP19*).^51^ qRT-PCR showed its expression peaked at 24 hours of cold treatment (29.17-fold), making it the most responsive gene. In rice, LIP19 activates cold-resistant genes like *COR15A* by forming heterodimers with *OsOBF1* to bind G-boxes.^51^ Thus, *ZlbZIP005* likely regulates flavonoid synthase (e.g., CHS) and proline synthase (P5CS) genes in *Z. latifolia* through similar mechanisms, participating in antioxidant and osmotic adjustment pathways.^48,49^

*ZlbZIP075* (orthologous to rice *OsbZIP71*): Mapped to the qLTG5.2 homologous interval on chromosome 5 (rice low-temperature germination rate QTL), it contains three exons and harbors both G-boxes and methyl jasmonate-responsive elements (CGTCA-motif) in its promoter. Its expression was transiently downregulated at 6 hours but continuously increased afterward, reaching 4.13-fold at 24 hours (Figure 7(b)), consistent with rice *OsbZIP71*’s function in regulating ABA signaling and promoting sugar translocation to pollen via heterodimers.^33^ Notably, ZlbZIP075 acquired an EAR repression domain (LxLxL motif) through gene duplication, while its homolog ZlbZIP100 retained a complete activation domain, suggesting they may, respectively, inhibit ABA signaling and activate metabolic pathways.^35,55^

*ZlbZIP084* (orthologous to rice *OsOBF1*): Located on chromosome 11 with three exons, it has the most intact Motif 3 serine phosphorylation sites among cold candidates. Its expression increased 4.10-fold at 24 hours, slightly lower than *ZlbZIP005* but still significantly higher than the control (Figure 7(b)). In rice, *OsOBF1* interacts more strongly with *LIP19* than with its own homodimers, suggesting *ZlbZIP084* may enhance cold signal transduction efficiency through heterodimer formation.^51^

Collectively, these genes exhibit temporal expression patterns, implying a staged regulatory model: *ZlbZIP005* is rapidly activated in the early stage to initiate flavonoid synthesis for ROS scavenging, followed by coordinated upregulation of *ZlbZIP075/ZlbZIP084* in the middle stage to integrate ABA signaling and osmotic adjustment, and sustained expression in the late stage to maintain cold-resistant metabolic homeostasis.^50^ This parallels the heterodimer-mediated cold gene regulation model in rice,^33,51^ but the higher density of G-box elements in *Z. latifolia* (e.g., five in *ZlbZIP075*) may confer more sensitive and efficient cold responses.^18^

The temporal expression characteristics of the three genes indicate that the cold response in *Z. latifolia* involves stage-specific regulation:

### 4.3. Expression Patterns of Key Genes and Construction of Cold Response Networks

Early stage: Rapid activation of *ZlbZIP005* dominates, likely initiating flavonoid synthesis to scavenge reactive oxygen species (ROS). Flavonoids play a critical role in antioxidant defense by neutralizing ROS generated under cold stress.^50^

Mid-stage: Synergistic upregulation of *ZlbZIP075* and *ZlbZIP084* integrates ABA signaling and osmotic adjustment. These genes likely modulate ion balance and stress-related metabolite accumulation through interactions with hormone pathways.^33,51^

Late stage: Expression levels decline but remain significantly above baseline, ensuring sustained metabolic homeostasis for long-term cold adaptation. This pattern maintains essential cold-resistant processes while avoiding energy waste.

This regulatory model shares similarities with rice, where bZIP genes form heterodimers to regulate cold-responsive genes.^33,51^ However, the high density of G-box elements in *Z. latifolia* (e.g., 5 G-boxes in *ZlbZIP075* promoter) may confer more sensitive and efficient cold responses by enhancing bZIP-DNA binding affinity.^18^

Gene duplication analysis revealed that segmental duplication was the primary expansion mechanism for the ZlbZIP family. While 78.5% of gene pairs were under purifying selection (Ka/Ks < 1), maintaining functional conservation, 15.7% (e.g., ZlbZIP005-ZlbZIP021, Ka/Ks = 1.23) experienced positive selection.^55^ This evolutionary pressure drove differential enrichment of cis-elements, creating a division of labor between “dominant regulation” and “functional redundancy” to enhance the robustness of cold response networks.^35^ For example, *ZlbZIP005* (with 5 cold-responsive elements) and its duplicate *ZlbZIP021* (2 elements) may exhibit divergent induction kinetics while maintaining overlapping functions, ensuring regulatory flexibility under fluctuating cold stress.

### 4.4. Perspective: Research Pathway from Molecular Mechanism Elucidation to Breeding Application

Aiming at the core regulatory functions of the *Z. latifolia* ZlbZIP family in cold response, future research can systematically advance the deep conversion of cold-resistant gene resources into crop improvement through an integrated pathway of “mechanism elucidation-network construction-breeding transformation.” At the molecular mechanism level, studies should focus on key genes in the ZlbZIP family to clarify their regulatory associations with flavonoid and proline metabolic pathways, reveal the effects of phosphorylation modifications on protein functions and their roles as low-temperature signaling molecules, and dissect their regulatory roles in hormone signaling networks such as ABA. This will lay a foundation for elucidating the molecular mechanisms of cold tolerance and establishing regulatory pathways from genes to traits. At the application level, constructing a “low-temperature signal perception-transcriptional regulation-metabolic response” network through multi-omics integration, combined with methylation sequencing and genome-wide association studies (GWAS), will help decipher epigenetic modifications and SNP loci related to cold tolerance. Gene editing technologies can be used to target rice homologous genes, overexpress related genes, or optimize G-box element density to create new cold-resistant germplasm. Incorporating bZIP family data from gramineous species for phylogenetic analysis, combined with Ka/Ks ratio analysis, will help identify positively selected genes and reveal the adaptive evolutionary trajectories of cold-resistant genes, providing multi-dimensional theoretical and technical support for crop stress-resistant breeding.

## 5. Conclusions

Based on this, 115 bZIP transcription factors (ZlbZIPs) were identified, with 18 genes located in known cold-resistant QTL intervals. Four cold-resistant candidate genes were screened through collinearity analysis with the rice genome. Phylogenetic analysis showed they share 10 subfamilies with rice but exhibit subfamily-specific expansion and contraction. 61.74% of genes contain 1–4 exons, and the core conserved motif Motif 1 is highly conserved across all subfamilies. Motif 3 carried by cold-resistant candidate genes may enhance transcription factor activity through phosphorylation modification.

qRT-PCR verification showed that *ZlbZIP005, ZlbZIP075*, and *ZlbZIP084* were significantly upregulated at 24 hours of cold treatment (29.17-, 4.13-, and 4.10-fold, respectively), and the high-density G-box elements in their promoter regions imply stronger cold-responsive potential. Gene duplication analysis indicated that segmental duplication was the main expansion mechanism, with 78.5% of gene pairs maintained for conservation through purifying selection and 15.7% experiencing positive selection to promote functional divergence.

This study fills the gap in cold metabolomics research on *Z. latifolia*. The identified key cold-resistant candidate genes may participate in cold-resistant pathways by regulating flavonoid synthases and proline synthases. Their high collinearity with rice provides excellent genetic resources and targets for crop cold-resistant breeding. Future integration of functional validation and multi-omics will provide precise theoretical guidance for deciphering plant cold resistance mechanisms and cultivating low-temperature-tolerant varieties, which is of great significance for food security under climate change.

## Supplementary Material

Supplemental Material

## Data Availability

The original contributions presented in the study are included in the article, further inquiries can be directed to the corresponding authors.
